# Camels, Camel Milk, and Camel Milk Product Situation in Kenya in Relation to the World

**DOI:** 10.1155/2022/1237423

**Published:** 2022-03-08

**Authors:** Stephen Oselu, Rebecca Ebere, Joshua M. Arimi

**Affiliations:** Department of Food Science, Meru University of Science and Technology, Meru, Kenya

## Abstract

Kenya is the leading camel milk producer globally, with an annual production volume of 1.165 MMT, followed by Somalia (0.958 MMT) and Mali (0.271 MMT). In Kenya, pastoral tribes in North-Eastern parts rear about 4.722 million camels accounting for about 80% of all camels. Camels offer locals various benefits, including transportation of goods across the deserts, meat, fur, and milk. Camel milk contains natural therapeutically and immunity-boosting properties due to the higher concentration of lactoferrin, lactoglobulins, and lysozyme than bovine milk. Camel milk has been shown to have hypoallergenicity properties compared to bovine milk. Camel and human milk are similar in nutritional composition and therapeutic properties. Camel milk is known to fight various diseases, including cancer, diabetes, autism, hypertension, and skin diseases. Despite the standing of Kenya in the world in terms of camel milk production, Kenya lags considering the camel milk products, industries, and marketing. This paper reviews recent literature on camels and camel milk production trends in Kenya in relation to the world. The review also discusses various camel milk properties (nutritional and therapeutic) as well as the camel milk sector situation in Kenya.

## 1. Introduction

Camels (*Camelus dromedarius*) are animals reared in the desert and were first domesticated in the Southern region of Arabia more than 4,500 years ago [1]. Camels were first used to solve transportation problems and offered locals meat, milk, fur, tourism, agricultural work, racing, and beauty contests [2, 3]. Therefore, camels play a great role in meeting subsistence needs in the Arid and Semi Arid Lands (ASALs) of Kenya. Within the ASALs, camels are majorly reared for milk production as they produce milk for extended periods compared to cattle especially in dry areas [4]. Camel milk provides nutrients and medicinal benefits to consumers ([5, 6]). During seasons of extended drought, camel milk contributes up to 50% of the total nutrient consumption among pastoralist communities in Northern Kenya, hence a critical factor in ensuring food security within Kenya's ASALs [7].

Over the decades, camel milk production in Kenya has been increasing since 2014 with a sharp rise between 2018 and 2019 [8]. Kenya is the current world's leading producer of camel milk followed by Somalia [8]. According to the global camel milk production rankings by FAOSTAT [8], Kenya produces about 1.165 million litres followed by Somalia and Saudi Arabia at 0.956 and 0.271 million tonnes, respectively. The increasing in camel milk production volumes has been due to the rising adoption of camel rearing practices, improved production techniques supported by various organizations, and improving camel milk consumption in Kenya.

Despite this ranking, the production and marketing of camel milk products in Kenya is still very low. About 50% of all the camel milk produced in Kenya goes to waste due to postharvest losses resulting from poor hygiene, lack of proper handling equipment, poor infrastructure, and long durations during transportation of camel milk at ambient temperatures which inturn harbor microbial growth and elevate milk deterioration [9, 10]. This paper reviews recent literature on camels and camel milk production in Kenya in relation to the global status. It also discusses camel milk benefits, both nutritional and therapeutic, as well as the situation of camel milk processing in Kenya.

## 2. Camel Population Globally and Camel Breeds in Kenya

### 2.1. Camels Population

According to FAOSTAT [8], the world's camel population stands at approximately 37.51 million, with 89.4% being one-humped dromedary camels (*Camelus dromedarius*) and 10.6% million two-humped camels (*Camelus bactrianus*) (Table 1).

Further, about 87.1% (32.7 million) of the world's camel population is in Africa, with 13.71 million camels in Eastern African countries, including Kenya, Somalia, Ethiopia, and South Sudan (Table 1). According to FAOSTAT [8], Kenya currently hosts approximately 4.721 million camels. Nevertheless, Kenya's North-Eastern counties, including Mandera, Wajir, Garissa, Turkana, Marsabit, Isiolo, Tana River, Samburu, Baringo, and West Pokot, host most camels in that order, with pastoral communities Turkana, Gabbra, Somali, and Rendile being the key players [11, 12]. Mandera, Wajir, and Garissa countries have the highest camel population in Kenya with about 1.83 million, 1.177 million, and 816 thousand camels, respectively (Table 2) [11].

In Kenya, various ASAL county governments and multiple development agencies like the Ewaso Nyiro Development Authority, FARM lands, Lands Resource Management, Arid Lands, CARE Kenya, Kenya Agricultural and Livestock Research Organization (KALRO), USAID, and Food for the Hungry (FH) promote the rearing of camels by Kenyan pastoralist communities [13]. Camels are known to contribute to food security in ASAL regions since cattle and other livestock cannot withstand the harsh climatic variability of ASAL areas compared to camels [14].

### 2.2. Camel Breeds in Kenya

Various camel breeds exist in Kenya and are classified based on specific geographical distribution and ethnic groups owning them [15]. The ASAL communities distinguish the camel breeds into four main groups: Turkana, Rendile, Gabbra, and Somali [15]. Also, an imported breed of Pakistani camels is reared in Laikipia ranches [16].

**Figure 1:**
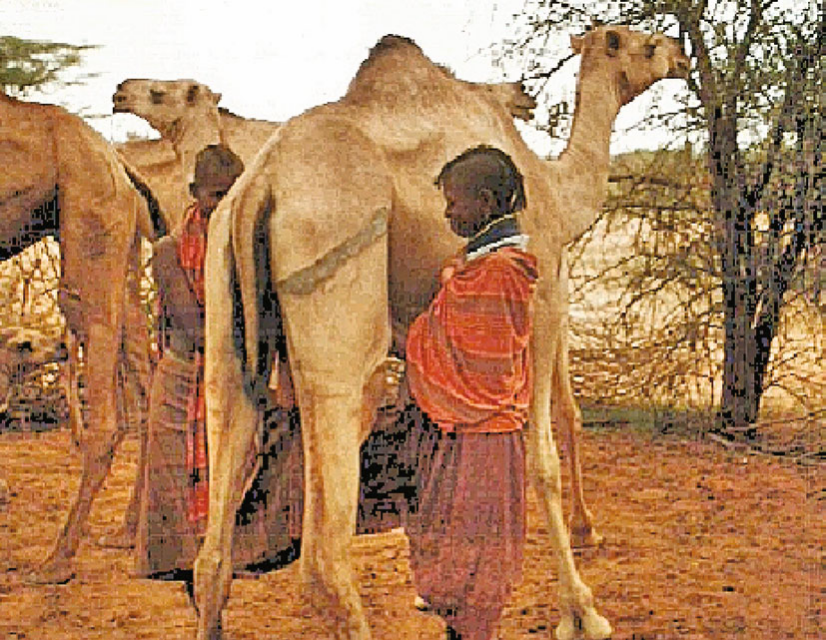
The Turkana breeds. Source: [17] *(with permission from authors).*

The Turkana breed is short with a dark brownish color [15] (Figure 1). The Turkana camel breeds weigh between 250 and 500 kgs (live weight) and have a standing height of about 1.7 meters when mature. These breeds are the most resilient and hardy compared to all other breeds in Kenya [15]. They can climb steep lava hills and are least affected by feed scarcity [16].

**Figure 2:**
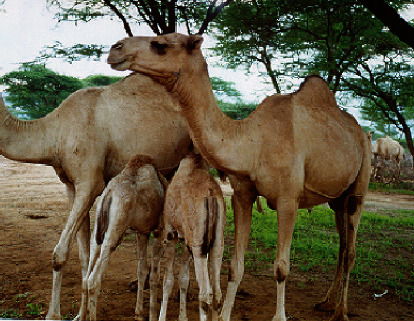
The Gabbra/Rendile breeds. Source: [17] *(with permission from authors).*

Figure 2 shows Rendile and Gabbra camel breeds. These breeds weigh about 300-550 kgs (live weight) with a standing height of 1.8 meters when mature. These camels do well under poor pasture conditions and rough terrain hence more tolerant to the drought conditions [15].

**Figure 3:**
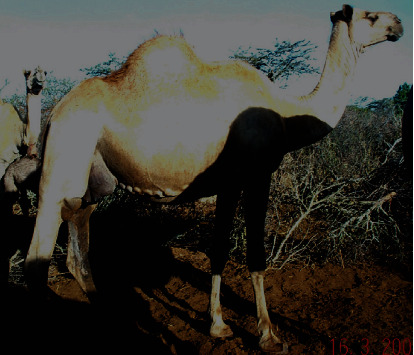
The Somali breeds. Source: [17] *(with permission from authors).*

According to Ogolla et al. [18], the Somali breed is white and tall, with male camels having a broader face (Figure 3). The Somali breeds weigh between 450 and 850 kgs with a height of about 2 meters when mature. Due to their large sizes, these camels cannot be reared in areas characterized by rough and rocky hillsides [15].

**Figure 4:**
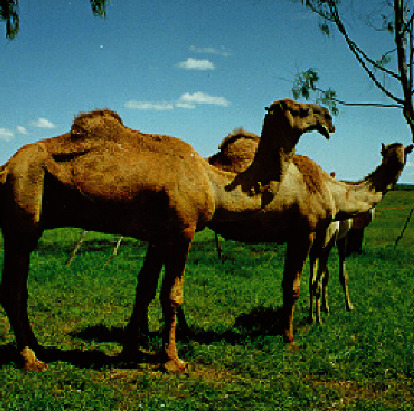
The Pakistani breeds. Source: [17] *(with permission from authors).*

The other group of camels consists of the Pakistani breeds (Figure 4) imported from Pakistan into ranches in Laikipia county in Kenya in the early 1990s [16]. When mature, the Pakistani breeds weigh between 400 and 700kgs (live weight) with a standing height of about 1.9 meters. Crossbreeds of Pakistani with Turkana and Somali breeds have since been distributed out of Laikipia county to other counties like Samburu, East Pokot, Kajiado, Mandera, and Marsabit [16].

## 3. Camel Milk Production among Breeds and the Production Trends

### 3.1. Milk Production among the Camel Breeds in Kenya

The Pakistan camel breeds produce the highest quantity of milk, with a daily production of between 4 and7 litres of milk under ranching conditions [15]. However, despite high milk yield, they rely on heavy feeder programs and cannot survive the harsh desert conditions and rough terrain witnessed in the North-Eastern counties in Kenya [16].

The Somali breeds are classified into four subtypes: Siftarr, Hoor, Gelab, and Aidimo [16]. The Hoor camels produce the highest quantities of milk [16]. The Somali camel breeds are the second-highest milk producers, producing about 3–5 litres of milk daily, with a lactation period between 12 and 18 months.

The Rendille and Gabra camels have a milk production yield of between 1 and 3 litres of milk a day and have a lactation period of between 12 and 18 months.

The Turkana camel breeds produce less milk than the Somali, Gabbra, and Rendille breeds, with a daily production of between 1 and 1.25 litres of milk. The Turkana breeds have a lactation period of about 12 months. Additionally, the Turkana breeds take a longer period of time before attaining maturity compared to other camel breeds [16].

### 3.2. Camel Milk Production Trends Globally and in Kenya

**Figure 5:**
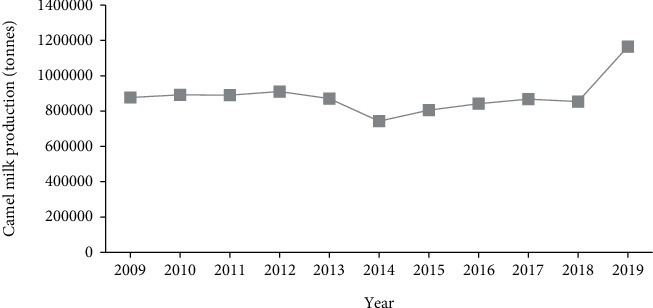
Camel milk production trend in Kenya for the past decade (2009–2019). Source: [8].

According to FAOSTAT [8], Kenya is currently ranked the highest camel milk producer in the world, followed by Somalia with approximately 1.165 million and 0.958 million tonnes, respectively (Table 3). The production of camel milk in Kenya has been on the rise in the past decade due to the simultaneous improvement of camel milk consumption, camel rearing, and camel milk marketing. According to reports from [8], annual camel milk production increased by about 36.24% (310,000 litres) from the average production of 855 thousand litres in the last decade (2009–2019) (Figure 5).

Camel milk productivity varies in different regions in Kenya depending on the availability of water, quality of feeds, camel breeds, camel-health status, and milking frequencies [19]. On average, camels produce six times more milk than the amount produced by indigenous cattle reared in drylands [13].

Kenyan population consumes milk primarily from cattle, goats, and camels that support about 1.8 million rural households and offers over 7 thousand job opportunities in the dairy sector [20]. Camel milk is essential in generating about 40.9% and 35.7% household income during wet and dry seasons, respectively [21].

During the camel lactation period (0–18 months), different camel breeds in Kenya produce between 1000 and 3,500 litres of milk, translating to between ~1.5 and 6 litres per day [16]. In Kenya, the locals usually consume the highest amount of milk produced. A small amount of milk is marketed in urban areas due to unhygienic milk handling and lack of efficient cooling facilities and skills for camel milk preservation along the supply chain.

Typically, Camel milk is primarily consumed in North Eastern counties at subsistence levels. Isako and Kimindu [22] indicate that camel milk contributes up to 50% of nutrient uptake during drought seasons due to the scarcity of milk from cattle, sheep, and goats. In Saudi Arabia, Qatar, and Oman, dairy farms have modern equipment for enhancing camel milk and meat production [23]. However, African countries, including Kenya, have limited mechanised camel milk production equipment.

## 4. The Nutritional Composition of Camel Milk

Camel milk is opaque white in colour with a slightly salty taste [24]. According to [19], camel milk comprises lactose, fat, and protein in an averagely similar manner to bovine milk (Table 4).

Camel milk protein concentration varies from 2.3% to 3.92% but does not contain beta-lactoglobulin [25]. Reports also indicate that camel milk contains a higher whey protein to casein ratio than bovine milk, providing a soft and easily digestible curd in the gastrointestinal tract [26]. Besides, camel milk casein is considered to be relatively larger with an average diameter of 380 nm compared to 150 nm (bovine), 260 nm (caprine), and 180 nm (ovine) milk. Glantz et al. [27] reckon that other livestock kinds of milk portray better gelation properties due to the presence of casein proteins of smaller sizes. In addition, camel milk also has protective proteins, mostly enzymes, which contribute to the antibacterial and immunological properties. These proteins include lysozymes (0.03–0.65 mg/dL), immunoglobulins, lactoferrin (95–250 mg/dL), and lactoperoxidase (2.23 ± 0.01 U/mL) [24].

In terms of fat composition, Singh et al. [23] report that camel milk fat is primarily composed of polyunsaturated fatty acids (PUFAs) and fewer short-chain fatty acids (C-4 to C-14) (Table 4). Camel milk is also known for its high antioxidant properties as it contains three times [30] to five times [31] higher vitamin C content compared to bovine milk. Dromedary camel milk contains an average of 34.16 mg/L vitamin C content [30, 32]. The high vitamin C (ascorbic acid) content lowers the pH in camel milk, hence stabilizing it and enabling it to stay longer without cream formation compared to milk from other livestock [33].

In terms of mineral composition, camel milk contains relatively more iron and calcium and has a high ascorbic acid content that boosts absorption in the duodenum [24]. Camel milk also contains high content of zinc essential for maintaining and boosting the immune system. The average concentrations of Ca, K, Fe, Zn, Na, and Cu are shown in Table 5.

## 5. Therapeutic Properties of Camel Milk

Despite camel milk not battling for the shelf space with cow milk in the dairy section of various Kenyan markets, it is becoming a product of interest among academicians and the general public due to therapeutical benefits [22]. Since ancient times, it has also been used as a nutraceutical and therapeutic product in the population of the Middle East and some parts of Africa and Asia [35]. Camel milk is documented to possess properties that fight various diseases, including cancer, diabetes, autism, hypertension, and skin diseases [24, 35, 36]. This section discusses various health benefits obtained from camel milk and its products.

### 5.1. The Antidiabetic Properties of Camel Milk

Studies have shown promising nutraceutical properties of camel milk that can boost diabetes management [35, 37–39]. Agrawal et al. [40] report that camel milk is safe and efficient for boosting long-term control of blood sugar levels, significantly cutting insulin doses for patients with type 1 diabetes.

In a study conducted in India, Abdalla [41] compared the conventionally treated juvenile diabetes with the population drinking camel milk. It was established that the camel milk-drinking group had significantly reduced HbA1C levels (the average blood glucose levels for the last two to three months) and lower blood sugar. Further, camel milk contains insulin with unique properties, enhancing immunomodulatory and regulatory functions on human cells [40]. According to Jilo and Jilo [42], insulin in camel milk hardly coagulates under the human stomach's acidic conditions compared to insulin from other mammals.

Furthermore, camel milk insulin is engulfed within micelles and protected from proteolysis in the upper gastrointestinal tract [42]. It is encapsulated in nanoparticles that make it easy for the human system to absorb and easily pass it to the bloodstream. Additionally, the antioxidant property of camel milk is key in inhibiting metabolic syndrome, which involves insulin resistance, hyperlipidaemia, and hyperglycaemia [43].

The property of camel milk in reducing the insulin requirement compared to bovine milk make it a perfect candidate for use as an adjunct to insulin therapy [40]. It is safe and efficient in improving long-term glycemic control, reducing insulin requirements in type 1 diabetic patients [40]. In support of these findings, several biochemical studies have shown that camel milk contains antimicrobial compounds, such as proteins, immunoglobulins, and lactoferrin, all of which are responsible for camel milk's antidiabetic properties [39]. Also, epidemiological surveys confirm low prevalence rates of diabetes among the camel milk-consuming population, indicating a hopeful function in controlling hyperglycemia [44].

Khan and Alzohairy [45] also conducted a long-term study to evaluate camel milk's safety, efficacy, and acceptability as an adjunct to insulin therapy in type 1 diabetics. This study compared the insulin needs for two groups of type 1 diabetic patients. The first group received conventional care: exercise, diet, and insulin, and the second group received regular care plus camel milk. Findings showed that the camel milk-receiving group had reduced mean blood sugar, haemoglobin, and insulin doses and eventually reduced insulin dose requirements to zero. The findings also showed a significant change in anti-insulin antibodies and plasma insulin in both groups. Therefore, Khan and Alzohairy [45] concluded that camel milk is safe and effective in enhancing long-term glycemic control among patients with type 1 diabetes.

### 5.2. The Antibacterial and Antiviral Properties of Camel Milk

Camel milk has been reported to be an excellent source of antimicrobial enzymes, including lactoperoxidase and lactoferrin, protective proteins involving small immunoglobulins compared to other ruminants [42]. The levels of lactoferrin and lysozyme are significantly higher in camel milk than in bovine milk [46]. Past studies have confirmed that lactoferrin functions as either a bactericidal and/or bacteriostatic agent [47]. This is why camel milk also has an inhibitory activity against Gram-positive and Gram-negative bacteria such as *Staphylococcus aureus, Escherichia coli*, and *Salmonella* Typhimurium [48].

On the other hand, Benkerroum et al. [49] report that camel milk has effective bacteriostatic impacts on *E. coli* and *L. monocytogenes*, while camel colostrum has bactericidal effects on *E.coli* and bacteriostatic effects on *L. monocytogenes.* Benkerroum et al. [49] compared the antimicrobial effects of raw camel milk and pasteurised milk. Raw camel milk has more effective antimicrobial properties, signalling that pasteurization destroys a portion of the antimicrobial compounds in camel milk [49].

A study conducted by Al-Majali et al. [47] also confirmed that camel milk lactoperoxidase has bacteriostatic effects against Gram-positive strains and bactericidal effects against Gram-negative cultures. The study also found that camel milk immunoglobulins have little impact on bacteria but contain elevated antibodies that fight rotavirus. Additionally, camel milk also has other antiviral characteristics hence playing a great role in improving the immune system in humans. El-Fakharany et al. [50] reckon that lactoferrin and immunoglobulin obtained from camel milk effectively inhibits the hepatitis C virus. These compounds have also demonstrated significant effects against synthetic peptides from camel milk [50]. Moreover, rotavirus is the most common cause of diarrhoea in children less than five years [51]. The high concentration of antirotavirus antibodies and the effective action of camel milk against rotavirus makes it essential in offering antidiarrheal/antibacterial properties, hence applied to manage diarrheal cases among the population.

### 5.3. Skin Disease Management Properties

The skin is the largest organ in the human body and is characterised by rapid growth compared to other organs. However, skin is exposed to a myriad of infections in people of all ages. Skin disorders are among the most irritating ailments that people can get accustomed to, specifically when the affected areas are around places hard to conceal even with makeup, for instance, the face or arms [52]. The skin problems become more worrying if the ailment is nonresponsive to skin disorder treatments. Camel milk is one of the solutions to this problem.

Camel milk has been proven to contain cosmetic effects due to the presence of *α*-hydroxyl acids known for plumping the skin and smoothing fine lines [42]. The *α*-hydroxyl acids in camel milk effectively shed the horny outer layer of dead skin cells by enhancing the breakdown of sugars that bind the skin cells together. As a result, new cells are revealed with elevated elasticity and clarity [42]. In a different study, *α*-hydroxyl acids from camel milk were found to eliminate wrinkles as well as age spots [42]. The *α*-hydroxyl acids also contribute to relieving dryness by making the outer layers of the skin thinner and supporting and thickening the inner layers of the dermis. Moreover, camel milk acts as a natural source of *α*-hydroxyl acids for softening the skin, keeping it healthy, smooth, supple, and with no wrinkles [53]. Additionally, the liposome found in camel milk is a potential cosmetic ingredient with antiageing properties [54].

Camel milk contains vitamin B, C, carotene, and iron vital for skin health [23]. Camel milk also contains lanolin and other moisturizing characteristics that provide a soothing effect on the skin [54]. Camel milk cosmetics have, therefore, been essential in keeping the skin beautiful and managing skin disorders such as eczema, acne, dermatitis, and psoriasis.

### 5.4. Anticancer, Antitumour, and Antiulcer Properties of Camel Milk

About 33% of cancer cases are potentially preventable in third-world countries, and another 33% are treatable when detected during early stages [55]. Camel milk, a highly nutritional and therapeutic product, has been shown to contain anticancer, antitumour, and antiulcer properties [36].

For instance, according to reports, lactoferrin in camel milk has inhibitory properties against the proliferation of cancer cells *in vitro* and repairs the damaged DNA tissues [56]. In support of these findings, Habib et al. [56] also confirmed that the main camel milk contains lactoferrin which acts as the main iron-binding protein and is responsible for 56% reduction of growth of cancerous cells and tissues. On the other hand, Korashy et al. [57] reckon that camel milk has also shown positive results in inhibiting the proliferation of human breast cells and minimises the rate of oxidative stress-mediated mechanisms.

Korashy et al. [57] also investigated the mechanisms that make camel milk becomes effective in controlling human cancer cells and concluded that camel milk induces apoptosis in human liver cancer cells—HepG2 and breast cancer cells—MCF7 through oxidative-stress-mediated and apoptotic mechanisms. Other studies also confirmed that camel milk has anticytotoxic and antigenotoxic impacts by inhibiting Micronucleated Polychromatic Erythrocytes (MnPCEs) and enhancing cells' mitotic index found in the bone marrow [58]. It has also been confirmed that camel milk effectively stops the growth of tumours and other malignant cells, including colon carcinoma, lung cancer cells, hepatocellular carcinoma, human glioma cells, and leukaemic cells (Gader and Alhaider 2016). Reports suggest that camel milk's anticancer properties result from either antiangiogenic (cutting blood supply to tumour cells) or direct cell cytotoxicity actions of camel milk lactoferrin (Gader and Alhaider 2016).

Camel milk contains very active antibodies that bind to tumour cells, destroying them without affecting healthy human tissues, mitigating the negative effects of tumours as human antibodies are too big to kill tumour cells [59]. Besides, Levy et al. [59] also reveal that camel milk contains strong antimicrobial and antioxidative activities that contribute to tumour growth. These properties also enhance the capacity of camel milk in reducing inflammation of the liver as it also contains nutrients that are essential for the healthy functioning of the liver [59].

Moreover, reports show that camel milk contains proteins with an antioxidative, angiotensin-converting enzyme (ACE) inhibitory, and antimicrobial characteristics [60]. According to Al-Ayadhi and Elamin [61], camel milk casein proteins show higher ACE inhibitory and antioxidant activities after enzymatic digestion [62]. Besides, cultured camel milk contains higher antioxidant and ACE-inhibitory properties than bovine milk due to the elevated amounts of proline and structural differences in camel milk caseins [63].

Camel milk has been proven to have antiulcer functions. Nonetheless, camel milk contains elevated amounts of vitamin A, C, B2, and E (acidic pH) and has high zinc and magnesium levels [64]. According to Traber and Stevens [65], these vitamins are vital in reducing the oxidative stress resulting from toxic agents, and magnesium is crucial for the absorption and metabolism of vitamins B, C, and tocopherol. Magnesium is also important in enhancing glutathione biosynthesis, preventing damage to cellular components caused by peroxides, heavy metals, and free radicals. Several studies show that magnesium in camel milk significantly boosts the antioxidant defence mechanisms in the body [66].

### 5.5. Autism Management Properties

Autism disease involves a group of complex disorders that affect the development of the brain. According to Chiarotti and Venerosi [67], the causes of most autism cases are primarily due to autoimmune disorders that affect intestinal enzymes responsible for forming amino acids from the milk protein casein. Mottron and Bzdok [68] claim that in some instances, casein is broken down to powerfull opioid casomorphine instead of mainly beta-lactoglobulin and beta-casein. This opioid is responsible for causing cognitive and behavioural symptoms that signal brain damage [69]. Yagil [70] also supports these findings by reiterating that cerebral symptoms result from the malfunction in which opioid casomorphine is formed instead of amino acids from cow milk caseins (beta-lactoglobulin and beta-casein), leading to autism.

However, camel milk lacks these two caseins that form casomorphine; hence, no symptoms develop [71]. Additionally, camel milk contains immunoglobulins responsible for initiating the immune system and nutritional benefits that enhance brain development [69].

Besides, Sharma and Singh [72] also confirm that camel milk has shown positive therapeutic results against autism. For instance, camel milk consumption among autistic children depicted a reduction in symptoms of autism. Camel milk consumption among these children also showed enhanced joint coordination, cognition, motor skills, language, and skin health [73]. Reports also show that camel milk decreases oxidative stress by altering enzymatic and nonenzymatic antioxidant molecules, which improve child autism in children as shown by the improved Childhood Autism Rating Scale (CARS) [61].

### 5.6. Antiallergic Properties of Camel Milk

In humans, milk protein allergies result mainly from beta-lactoglobulin in cow and mare milk [74]. Further, the presence of beta-casein in cow milk leads to hypersensitivity in humans [75]. However, camel milk does not cause allergies as it lacks beta-lactoglobulin. Besides, the beta-casein in camel milk has a different structure where camel milk contains A2 beta-casein while a higher portion of bovine milk consumed contain A1 beta-casein [76]. This phylogenetic variation makes it difficult for the circulating monoclonal antibodies and immunoglobulins to recognize them within the human system. In a review conducted by Gizachew et al. [77], children with chronic allergies to bovine milk improved rapidly after consuming camel milk. Therefore, camel milk may be considered an alternative protein source for nutrition in children allergic to bovine milk.

In support of these findings, El-Agamy et al. [74] also confirm that people allergic to bovine milk showed allergic reactions after consuming milk from other livestock, including buffalo, horse, sheep, and goat milk, because of the positive immunological cross-reaction, but studies confirmed that camel milk lacks allergens found in ruminant milk. Therefore, camel milk has immunoglobulins resembling human milk, which minimises allergic reactions while strengthening their future response to foods. Therefore, camel milk can be used as an alternative for human milk for children and individuals experiencing milk protein allergy from ruminant milk [78–80].

## 6. Processed Camel Milk Products

Kenya is the highest camel milk producer in the world, it would be expected that the camel milk industry would be very well developed. However, this is not the case [22]. In Kenya, there are various camel milk products in the market. These products include whole pasteurised camel milk [10, 18]. Suusa is an indigenous product from raw camel milk produced through spontaneous fermentation, especially by pastoralist communities of North-Eastern Kenya [81]. On the other hand, camel milk fermented products are limited due to the challenges experienced during the production processes. These products include camel yoghurt, camel cheese, and butter [12].

### 6.1. Fresh Unpasteurised Camel Milk

Camel milk is among the primary sources of income, food security, and a significant cultural function among pastoral communities [21]. Camel milk acts as the essential diet for pastoralists as it contributes to about 50% of the total nutrient intake as well as 30% of annual caloric intake [10]. According to Muloi et al. [82], about 80% of the camel milk produced among Kenyan pastoralist communities is consumed raw and/or for making tea. Isako and Kimindu [22] observed that most of the camel milk is consumed raw due to its long standing cultural beliefs that it cures malaria, jaundice, and other diseases. Surplus camel milk is typically sold in urban centres, hence generating income for various pastoral households.

Despite the merits of camel milk among pastoralists, it still faces various problems such as high postharvest quantity losses and quality deterioration. As a result, in Kenya, about 50% of camel milk produced is wasted [9, 10]. According to Odongo et al. [10], the risk factors leading to high postharvest losses are associated with the camel milk value chain. These factors render camel milk unfit for processing and human consumption: most importantly, poor personnel hygiene, inadequate cleaning, lack of potable water for cleaning of milk containers, unfavourable high ambient temperatures, lack of knowledge and skills for camel health management, long time taken before camel milk is cooled, and poor cleaning and sanitation of camel milk containers [10, 18, 83].

Moreover, camel milk farmers and traders encounter additional economic challenges due to spoilage, delayed payments, financial losses due to informal courier, low prices of camel milk fermented products, as well as rejection of camel milk by customers in urban markets such as Nairobi [4]. Milk spoilage is the most predominant challenge as camel milk is obtained from various farmers, power losses in the cooling systems, mixing of camel milk obtained at different times (morning and evening milk) and the long distances between manyatta and urban centers as well as the exposure of milk to relatively high ambient temperatures, and use of dirty plastic containers [4, 84].

### 6.2. Pasteurised Camel Milk

Camel milk pasteurization is conventionally conducted using standard techniques used for cow's milk among camel producing countries [85]. However, among ASAL communities in Kenya, camel milk is boiled for preservation purposes due to the lack of improved heating techniques [84]. The standard pasteurization methods used include 60°C for 30 min [86], 75°C for 15 s [28], and 63°C for 30 min [87]. In some studies, camel milk portrays various properties during heat treatment. For instance, camel milk gives a significant amount of dry deposit on a stainless-steel plate during pasteurization between 60 and 90°C for about 1 to 2 hours [88]. According to Konuspayeva and Faye [83], these deposits originate from camel milk proteins due to the lower quantities of free thiol groups compared to bovine milk.

Camel milk whey proteins are also denatured under temperatures beyond 63°C and are specifically evident at 98°C [89]. Therefore, camel milk could produce elevated amounts of “milk stones,” and hence, the deposit milk residues can accumulate in insufficiently cleaned processing equipment, harbor more spoilage bacteria, and contribute to bad flavours in milk [83].

### 6.3. Ultraheat Treated Milk (UHT milk)

Many milk producers and retailers reap massive benefits provided the products does not need refrigeration and can be stored for an extended period within the shelves. For instance, Elhadi et al. [21] reckon that milk producers can serve wider markets, make production planning simpler through minimising product changes and losses, deliver milk and milk products with ease using fewer and cheaper distribution means, and lower the rates of product returns. Besides, retailers also make surplus profits as they do not have to procure expensive refrigerated display spaces [22].

However, sterilization of camel milk through UHT treatment is not yet achieved even if private institutions are working hard to develop the most effective technique. During UHT treatment of camel milk, milk processors face challenges due to the heat resistance of casein and whey proteins, vitamins, fat globules, or other compounds inherent in camel milk [90]. Supporting these findings, Momen et al. [91] also found that UHT treatment denatures whey proteins due to their oversensitivity to high heat conditions. However, to stabilize these proteins after UHT treatment, Alhaj et al. [92] acknowledge that adding chemicals (calcium chloride, ethylenediaminetetraacetic salt, k-casein from cow, sodium hydroxide, and sodium dihydrogen phosphate anhydrous) has been effective despite the need for further research before full commercialisation of UHT camel milk [92]. Currently, these challenges have made UHT processing in Kenya impossible [84].

### 6.4. HTST Processed Milk

HTST or High-Temperature Short Time Pasteurization is an essential milk processing technique today. HTST is also referred to as flash pasteurization. It is used during milk pasteurisation to destroy pathogenic microbes, including bacteria, moulds, yeasts, viruses, and protozoa, in milk products to ensure the production and sale of safe products to the public [93]. In Kenya, most companies pasteurize milk using conventional methods by heating milk to about 90°C for between 15 and 30 minutes [82, 84].

According to Stabel and Lambertz [85], HTST is done at 71.7°C for 15 secs, resulting in 99.9999% or greater reduction in harmful bacteria. HTST has been proved to be more energy-efficient than batch pasteurization, as the energy from the pasteurized milk can be applied in preheating raw milk. Furthermore, HTST being a continuous process handles higher volumes of milk in the industry. However, HTST is characterised by cooked flavour compared to batch pasteurisation in camel milk processing [93].

### 6.5. Companies Processing Fresh Camel Milk in Kenya

Today, public interest in camel milk and related products is increasing, especially in the Horn of Africa, including Kenya. This is due to the effects of drought and climate uncertainties threatening the livelihoods of ASAL communities, especially women, children, and the youth [22]. In this regard, the Kenyan government, NGOs, and the private sector continue to recognise the camel industry's strategic importance and support its growth.

In a study conducted by Siloma [94], The Anolei Women's Camel Milk Cooperative is among the biggest producers of camel milk in Kenya. The business is headquartered in Isiolo Town. Anolei Women's Camel Milk Cooperative processes between 2,500 and 4,200 litres of camel milk per day during dry and wet seasons, respectively. According to a publication by Africa Harvest [95], the organisation initially transported its final products to Nairobi using public transport to ferry about 3000 litres of camel milk daily.

The White Gold-Ngamia Milk processor also works on bringing camel milk to the consumers. The company is located in Thingithu estate in the outskirts of Nanyuki town, Laikipia county. White Gold-Ngamia milk has a processing capacity of 700 litres of camel milk daily. However, the company is currently pasteurizing and packaging less than 200 litres of camel milk daily [96].

Other companies that process camel milk include Nuug Camel Milk Products Ltd, located in Industrial Area in Nairobi [97]. The company collects, pasteurises, and packages camel milk from different ASAL areas in North-Eastern Kenya [97]. In Moyale, a company known as Moyale Camel Milk Dairy Cooperative Society was initiated in 2018 by a group of 20 camel milk farmers [98]. The cooperative currently processes pasteurised camel milk and sells its products in Moyale, Marsabit, and other parts of the country. Herders supply the firm with 2,000 to 3,000 litres of camel milk daily [98]. Moyale Camel Milk Cooperative Society has refrigerated milk handling facilities to improve the shelf life of camel milk. However, the cooperative lacks transportation facilities like vans for picking and delivering milk to designated towns and collection points [98].

### 6.6. Challenges Affecting Camel Milk Production in Kenya

Despite efforts by various organisations, agencies, and the Government of Kenya to support camel milk farming and processing through improving production technologies and information packages in the North-Eastern counties, sustaining these initiatives has been a great challenge [84]. According to Wayua and Adongo [84], KARI's introduced technologies collapsed due to several reasons. For instance, the project created awareness, training, new production technologies and equipment, and funding opportunities for farmers. However, despite the communities appreciating these appropriate modern technologies which they can operate on their own and use to generate income, sustaining them proves impossible as they are slow to adapt to the new techniques, negatively affecting camel milk technology-development efforts [84].

To sufficiently upgrade the camel milk industry in Kenya, there is a need to demonstrate what the camel milk means as a business venture. Besides offering strategic guidance and support, the sector requires proper investments for the industry to blossom. However, insecurity in Northern Kenya has been a great setback for different camel milk initiatives, production, and marketing [10, 20].

## 7. Camel Milk Butter Processing

Camel milk butter is primarily white with a more dense consistency than bovine milk butter [99]. In some pastoral communities, such as the Sahara region, camel butter is produced in small amounts for medicinal uses [100]. According to Konuspayeva et al. [101], camel milk fat content ranges between 1.2 and 6.4%, which is comparable to the fat content of bovine milk. Nonetheless, butter is not a traditional product manufactured from camel milk in Kenya [102]. Further, camel milk butter has been difficult to produce using traditional production methods used for bovine milk due to its different composition. However, according to Farah [102], camel milk butter is only produced in camel milk industries based in Nairobi. Generally, butter in Kenya is considered a luxury product although it could be of great nutritional importance. Besides, the relatively higher melting point of camel milk fat (41–43°C) increases the difficulty of processing camel butter at temperatures between 10 and 14°C, which is the optimum temperature for churning bovine milk.

The processing of camel milk into butter is a challenging process due to its slight tendency to cream up because of the lack of the protein agglutinin, small fat globule sizes, as well as the thicker fat globular membranes [5, 103, 104]. On the other hand, camel milk contains a lower concentration of short-chain fatty acids and higher amounts of long-chain fatty acids [105]. The high amounts of long-chain fatty acids in the fatty-acid profile of camel milk could be the main contributor to camel milk's high melting point.

However, several studies highlight the possibility of producing camel butter under optimum churning conditions of agitation and temperature [19, 106, 107]. According to Berhe et al. [19], vigorous agitation and shaking of fermented camel milk in a vertical position instead of the back and forth-agitation method at relatively higher churning temperatures of about 22–23°C could produce camel butter with 80% fat recovery efficiency. This vigorous method exerts an additional force that raptures the fat globule membrane and allows the fat globules to coalesce to one another. Similarly, camel butter was produced by Mtibaa et al. [108] using temperatures of between 15–36°C with the highest butterfat recovery (85%) at a churning temperature of 25°C.

## 8. Camel Milk Cheese Production in Kenya

### 8.1. Camel Milk Cheese Processing

Traditionally, pastoral communities typically consume camel milk while fresh or fermented [22]. The development of fermented camel milk products continues to increase despite the challenges that hinder the fermentation of products such as cheese or yoghurt [109]. However, studies show that it is possible to manufacture cheese from camel milk [23]. The primary challenges in producing fermented products from camel milk involve its unique functional and structural characteristics. Ramet [107] reports that camel milk contains low amounts of kappa casein, leading to a weak network of caseins easily broken in the cutting process stage during cheese production. This challenge results in the massive loss of dry matter when whey is drained during the cheese-making process.

Compared to bovine milk, camel milk contains 26% more beta-casein (*β*-CN) in a specific volume of milk. Camel milk also has low kappa-casein (3.5%) than bovine milk (13%). On the other hand, the amount of kappa-casein in camel milk is considered less, contributing to its coagulation challenges in cheese making [5]. However, Berhe et al. [105] confirm that milk coagulation is obtainable through enzymatic hydrolysis of kappa-casein at the surface of casein micelles.

Camel milk comprises alpha-lactalbumin as the primary protein, as in the human milk whey protein group. Numerous studies cite a high whey protein to casein ratio in camel milk compared to bovine milk [19, 99, 110, 111]. This is the main reason why camel milk contains a soft and easily digestible curd in the human gastrointestinal tract [26].

Regarding the sizes of casein micelles, camel milk contains casein relatively larger casein micelle diameters (380 nm) compared to that of bovine, ovine, and caprine milk with 150, 180, and 260 nm, respectively [112]. According to Glantz et al. [27], bovine milk portrays better gelation properties due to its smaller casein micelles. In summary, the high whey to casein protein ratio, lower levels of kappa-casein, and the larger micelle size associated with camel milk are responsible for the difficulty of camel cheese production [19, 99]. These camel milk properties lead to the formation of a weak coagulum and lower yield during camel cheese making [112, 113].

### 8.2. Status of Camel Milk Cheese Processing in Kenya

Despite numerous interventions to strengthen value addition processes to camel milk products, these challenges within the remote rangelands have rendered making camel butter and cheese impossible in most pastoral systems [114]. Furthermore, camel cheese and butter processing is not a norm among the pastoral communities in Kenya as these products are obtained from other livestock like cows, sheep, and goat milk [115, 116].

According to a study conducted in Oleleshwa Entreprises Limited (2014), camel milk cheese does not work well with mesophilic cultures. They result in a final cheese product characterised with an unpleasant mouthfeel [117]. On the other hand, thermophilic and mozzarella cultures produced better results based on camel milk cheese's acceptability and overall quality [117]. Commercial starter cultures are dispatched in sealed freeze-dried pouches and must be kept in deep freezers. Contrary, the ASAL pastoralist areas lack deep freezers, hence not possible to make various batches of camel cheese using commercial cultures [117].

Due to camel milk being more resistant to fermentation than milk from other livestock (cow or goat milk), Bruntse [117] speeded up the camel cheese process through the application of direct vat set (DVS) cultures to produce mother cultures a day before cheese production. DVS cultures are directly used to start the fermentation of milk [118]. To produce mother cultures, about half a litre is boiled and then cooled to 45°C. A small pinch of DVS cultures is added then kept under warm conditions until the next day for the production of camel milk cheese.

In some cases, the locals use “kibui cultures” to make camel milk cheese. The “Kibui” are the milk containers used by pastoralists to store camel milk [117]. They are made of either wood or leather, coated with colostrum for waterproofing and rinsed and smoked for cleaning and sterilisation after using them to carry or store milk. Over time, these containers produce very good fermented milk, a characteristic applicable in the production of camel milk cheese culture [117].

## 9. Camel Milk Yoghurt Production in Kenya

### 9.1. Camel Milk Processing

The processing of set-type yoghurt faces similar problems as cheese because the camel milk coagulum lacks the desired firmness and formation to produce yoghurt of desired texture and mouthfeel [119]. Attia et al. [120] also confirmed that camel milk yoghurt is fragile, heterogeneous, and contains dispersed flakes. Therefore, the primary problems associated with camel yoghurt involve its weak texture and thin consistency [121]. According to Galeboe et al. [122], the texture of yoghurt is an essential characteristic that influences the mouthfeel, appearance, and overall acceptability. Besides, several studies have cited the possibility of the growth of commercial starter cultures in camel milk despite displaying lower acidification rates than bovine milk [19, 123, 124].

Nonetheless, several studies have indicated the possibility of camel yoghurt production [99, 125–127]. Hashim et al. [126] reckon that camel yoghurt firmness could be enhanced by supplementing camel milk with stabilizers like alginate, gelatin, and modified starch when incorporated with a calcium salt. However, Al-Zoreky and Al-Otaibi [99] indicated that the supplementation of camel milk with stabilizers could not enhance the consistency of camel milk yoghurt.

On the other hand, Ibrahim [128] argues that applying exopolysaccharide producing starter cultures could enhance the textural properties of camel yoghurt compared to conventional additives and stabilizers. Other reports also indicate a possibility of improving camel milk yoghurt texture through mixing camel milk with milk from other livestock such as ovine and bovine milk [37, 104, 129].

### 9.2. Status of Camel Milk Yoghurt Processing in Kenya

In Kenya, the production and sale of camel milk yoghurt have been limited due to its technical problems and low consumer acceptability [82]. Pasteurised whole milk is the main processed camel milk product comprising about 85% of total processed milk, followed by camel milk yoghurt at 7% [82]. In an article published by Business Daily (1^st^ July 2019), the Nuug Camel Milk Products started producing camel milk yoghurt through producing three flavours, strawberry, mango, and vanilla [97]. In addition, Isako and Kimindu [22] report that Tawakal Cooperative Society contributes to camel milk value addition by processing camel milk yoghurt and a local fermented sour product known as “Susa.” However, these companies no longer produce camel milk yoghurt due to challenges of achieving viscosity similar to bovine milk that customers are used to [22, 130].

## 10. Conclusion

Kenya is currently the highest camel milk producer with an annual production of about 1.165 MMT. Camel milk is mostly consumed raw or sour following spontaneous fermentation. Camel milk contributes 50% of nutrients among the desert locals in Kenya. Despite being used as a source of nutrients and a medicinal product, about 50% of camel milk goes to waste due to low hygiene levels, long distance transportation of milk at ambient temperatures, lack of milk handling equipment, and poor infrastructure. Proximate composition data show a more remarkable similarity between camel milk and human milk, hence an adequate substitute for children allergic to bovine milk protein. Studies cite camel properties that may fight various diseases, including cancer, diabetes, autism, hypertension, and skin diseases. As a result, many traders, researchers, and academicians refer to camel milk as the “Desert White Gold.”

The Kenya camel milk industry is nascent with very promising potential and lacks improved facilities to handle and preserve camel milk for further processing. The difficulty experienced in manufacturing camel milk products is mainly due to its unique functional characteristics. Therefore, producing traditional dairy products by applying similar technologies for bovine milk products leads to processing challenges. There is a need for concerted effort in research to develop camel milk products and market them as “camel milk products” without expecting the properties of the products to have similar properties to bovine milk products.

## Figures and Tables

**Table 1:** Approximate camel population in different countries (2019).

	Area	Value (Heads)
1	World	37509691
2	Africa	32671288
3	Eastern Africa (Kenya, South Sudan, Somalia, and Ethiopia)	13706206
4	Somalia	7243792
5	Sudan	4895000
6	Kenya	4721900
7	Ethiopia	1281468
8	Mali	1241093
9	Eritrea	388152
10	Djibouti	70894

Source: FAOSTAT [8].

**Table 2:** Camel population in selected counties in Kenya.

	County	Number of camels
1	Mandera	1828665
2	Wajir	1176532
3	Garissa	816057
4	Turkana	261923
5	Marsabit	215234
6	Isiolo	148859
7	Tana River	53298
8	Samburu	48172
9	Baringo	38500
10	West Pokot	19389
11	Laikipia	7827
12	Meru	5732
13	Kitui	5202
14	Kajiado	3584
15	Taita Taveta	2630

Source: [11].

**Table 3:** Comparison of camel milk production among countries.

	Country	Production (tonnes)
1	Kenya	1,165,210
2	Somalia	958221
3	Mali	270995
4	Saudi Arabia	135473
5	Sudan	62000
6	United Arab Emirates	54715
7	Algeria	14784
8	Eritrea	12364
8	Yemen	9751
9	India	6944
10	Tunisia	1095

Source: [8].

**Table 4:** Comparison of proximate composition of milk from different species.

Species	total solids (%)	Fat (%)	Protein (%)	Lactose (%)	ASH (%)
Camel	12.00	3.50	3.10	4.40	0.8
Bovine	12.70	3.70	3.40	4.80	0.7
Caprine	12.30	4.50	2.90	4.10	0.8
Ovine	19.30	7.40	4.50	4.80	1.0
Human	12.20	3.80	1.00	7.00	0.2

Source: ([28]; [29]).

**Table 5:** Camel milk mineral concentration in milliequivalents per litre (mEq/L), and milligrams per deciliter (mg/dL).

Mineral	Early lactation	Late lactation
Na	29.70 ± 0.53 mEq/L	35.49 ± 0.89 mEq/L
K	50.74 ± 0.51 mEq/L	71.86 ± 1.43 mEq/L
Ca	94.06 ± 0.75 mg/dL	97.32 ± 0.51 mg/dL
P	41.68 ± 0.55 mg/dL	47.14 ± 0.52 mg/dL
Mg	11.82 ± 0.22 mg/dL	13.58 ± 0.31 mg/dL
Fe	1.00 ± 0.12 mg/dL	—
Zn	2.00 ± 0.02 mg/dL	—
Cu	0.44 ± 0.04 mg/dL	—

Source: ([34]; [23]).

## Data Availability

Data are available from the authors, official company websites, and various Uniform Resource Locators (URLs), as listed in the references section. The data that support the findings of this study are available from the corresponding author, J.M, upon reasonable request.
